# Local Retail Food Environment and Consumption of Fruit and Vegetable among Adults in Hong Kong

**DOI:** 10.3390/ijerph15102247

**Published:** 2018-10-14

**Authors:** Ting Zhang, Bo Huang

**Affiliations:** 1 Institute of Space and Earth Information Science, The Chinese University of Hong Kong, Hong Kong, China; Tingzhang@link.cuhk.edu.hk; 2 Department of Geography and Resource Management, The Chinese University of Hong Kong, Hong Kong, China; 3 Shenzhen Research Institute, The Chinese University of Hong Kong, Shenzhen 518057, China

**Keywords:** geographic information systems, density, retail food outlets, accessibility

## Abstract

Outside of western countries, the study of the local food environment and evidence for its association with dietary behavior is limited. The aim of this paper was to examine the association between the local retail food environment and consumption of fruit and vegetables (FV) among adults in Hong Kong. Local retail food environment was measured by density of different types of retail food outlets (grocery stores, convenience stores, and fast food restaurants) within a 1000 m Euclidean buffer around individual’s homes using a geographic information system (GIS). The Retail Food Environment Index (RFEI) was calculated based on the relative density of fast-food restaurants and convenience stores to grocery stores. Logistic regressions were performed to examine associations using cross-sectional data of 1977 adults (18 years or older). Overall, people living in an area with the highest RFEI (Q4, >5.76) had significantly greater odds of infrequent FV consumption (<7 days/week) after covariates adjustment (infrequent fruit consumption: OR = 1.36, 95% CI 1.04–1.78; infrequent vegetable consumption: OR = 1.72, 95% CI 1.11–2.68) in comparison to the lowest RFEI (Q1, <2.25). Highest density of fast food restaurants (Q4, >53) was also significantly associated with greater odds of infrequent fruit consumption (<7 days/week) (unadjusted model: OR = 1.34, 95% CI 1.04–1.73), relative to lowest density of fast food restaurants (Q1, <13). No significant association of density of grocery stores or convenience stores was observed with infrequent FV consumption regardless of the covariates included in the model. Our results suggest that the ratio of fast-food restaurants and convenience stores to grocery stores near people’s home is an important environmental factor in meeting fruit and vegetable consumption guidelines. “Food swamps” (areas with an abundance of unhealthy foods) rather than “food deserts” (areas where there is limited access to healthy foods) seems to be more of a problem in Hong Kong’s urban areas. We advanced international literature by providing evidence in a non-western setting.

## 1. Introduction

Fruit and vegetable (FV) are important components of a healthy diet, and their sufficient daily consumption (at least 400 g; ~5 portions per day) could help prevent major, non-communicable diseases, such as cardiovascular diseases, heart disease, stroke, cancer, diabetes, and obesity, according to the World Health Organization [1]. Approximately 16.0 million (1.0%) disability adjusted life years (DALYs, a measure of the potential life lost due to premature mortality and the years of productive life lost due to disability) and 1.7 million (2.8%) of deaths worldwide are attributable to low fruit and vegetable consumption. Moreover, insufficient intake of fruit and vegetables is estimated to cause around 14% of gastrointestinal cancer deaths, about 11% of ischaemic heart disease deaths, and about 9% of stroke deaths globally [2]. It is critical to understand the determinants of FV consumption so that effective interventions could be taken for promoting a healthy diet. Although lack of intervention evidence [3], population-targeted approaches especially changing food environment combined with individual-level interventions are considered to have more potential to achieve larger and long-term reductions in the disease burden attributable to insufficient FV intake, compared to merely individual change [4]. There is growing interest in the role of the environment in promoting or hindering healthy eating [5].

The retail food environment (RFE) has recently received a particular focus from researchers and numerous agencies, such as WHO and Centers for Disease Control and Prevention (CDC), for population-wide improvements in eating and health. The RFE is characterized both by the ‘community nutrition environment’ (the local opportunities to acquire food) and ‘consumer nutrition environment’ (the environment within and around food environment) [6]. With the development of geographic information systems (GIS), GIS-based measures of spatial access to food outlets become dominant in food environment assessment [7,8]. Results from studies examining the association of food accessibility with diet quality or diet-related health outcome are less consistent [9,10], possibly because of different methods for classifying, locating, and analysing food stores [10]. However, there is some evidence to suggest that residents who have better access to healthy food outlets, such as supermarkets [11], fresh food stores [12], and street markets [13], tend to have healthier diets and lower levels of obesity. Alternatively, people with more access to unhealthy food outlets, such as convenience stores [14] and fast-food restaurants [15,16], tend to be obese. Thus, the location, number and type of food outlet may contribute to the obesogenic environment.

Specifically, studies in California [17] and Canada [18] found significant association between the Retail Food Environment Index (RFEI), calculated as a ratio of the count of fast-food outlets and convenience stores to supermarkets and produce vendors around respondents’ home, and obesity. The RFEI was constructed by the California Center for Public Health Advocacy and is a measure of relative prominence of food outlet types which allows for easier comparison between areas using a single food environment scores. Researchers found the lower the RFEI, the lower the odds of being obese. Such results indicate that the relative density of different food outlets may be an important component of food environments. The Centers for Disease Control and Prevention (CDC) also developed a modified Retail Food Environment Index (mRFEI) in 2011 that gauges healthy food provision based on the percentage of healthy food retailers within census tracts [19,20].

However, most studies on the relationship between food environment, diet and obesity have been conducted in western settings, primarily North America, the UK, and Australia [18,21,22]. Outside of the western countries, the evidence is limited and equivocal. Assumptions and measures about food environment in western settings may not apply in other societal context. For example, in a study of older Japanese individuals, researchers found that better access to supermarkets was related to higher BMI. Better access to fast food outlets or convenience stores was also associated with higher BMI, but only among those living alone [23]. Besides, Murakami et al. [24] found no significant independent association between neighbourhood store availability for meat, fish, fruit and vegetables, and rice and intake of each food in a group of young Japanese women. These findings suggest the need to develop culture-specific approaches to characterizing neighbourhood food environment. Hong Kong provides a reasonable study region in non-western settings. In Hong Kong, there is also an urgent need for food environment assessment due to high population prevalence of obesity. In 2016, 38.8% of the population aged 18–64 in Hong Kong were classified as overweight or obese (BMI ≥ 23.0), including 20.7% as obese [25]. The fast living rhythm in Hong Kong makes a growing number of people prefer unhealthy processed food. It is still unknown that whether the local retail food environment in Hong Kong affects individual FV consumption and how the different types of food outlet affect FV consumption.

Therefore, the purpose of this study was to examine the association between local retail food environment and FV consumption in a Hong Kong’s context. Using cross-sectional data of 1977 adults (18 years or older), we explored the association of the density of different types of retail food outlets (grocery stores, convenience stores and fast food restaurants) and relative density of fast-food restaurants and convenience stores to grocery stores RFEI within a 1000 m Euclidean buffer around people’s home with infrequent consumption of fruit or vegetable in Hong Kong. We hypothesized that residents of areas with high grocery stores density, low convenience stores density, low fast food restaurants density or low RFEI would be more likely to meet FV consumption recommendation.

## 2. Methods

### 2.1. Research Workflow

**Figure 1:**
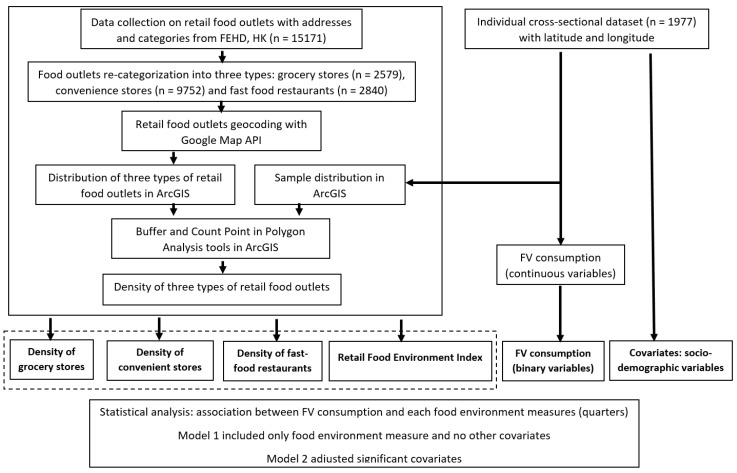
Research workflow.

Figure 1 provides a schematic representation of the research workflow. The process begins with two datasets for Hong Kong: an individual cross-sectional survey dataset and a dataset on retail food outlets. The workflow process includes several important steps undertaken for conducting this research. The most critical part is to characterize the local retail food environment by four food environment measures: density of grocery stores, density of convenient stores, density of fast-food restaurants, and RFEI. After four food environment measures were calculated for each respondent, together with FV consumption and socio-demographic variables, statistical analysis was performed to explore associations between FV consumption and local retail food environment. Details about each process are discussed in the following subsections.

### 2.2. Study Sample and Individual Measures

The individual cross-sectional survey data were from the first wave of the Strategic Public Research (SPPR) project—‘Trends and Implications of Poverty and Social Disadvantages in Hong Kong: A Multi-disciplinary and Longitudinal Study’ (http://www.poverty.hk) [26]. Face-to-face interviews were undertaken with 1980 adults (aged 18 or over) between May 2014 and July 2015. The sample was drawn from random addresses taken from the 2011 population Census. In this article, a dataset (*n* = 1977) with detailed home position information was used for our study.

FV consumption frequency data of respondents were collected in the cross-sectional survey. Respondents were asked “How many days a week do you usually have: (1) fruits; (2) vegetables”. Response options were continuous number from 1 to 7 (days). For analysis here, we dichotomized the outcome: infrequent consumption (<7 days/week) and frequent consumption (=7 days/week) since daily FV consumption is recommended by WHO. The following socio-demographic variables were also collected for covariates analysis: age, gender, marital status, house type, self-reported poverty level (“Do you think you are poor now?”), monthly income, education attainment, birth place, whether having under school age children.

### 2.3. Food Environment Measures

For characterizing the local food environment, we used three types of retail food outlets: grocery stores (including supermarkets, street market, and other fresh provision shops), convenience stores and fast-food restaurants. The community food environment can be measured by different spatial metrics, including density and proximity, and can be categorized into five types of geographic food access measures: container, container buffer, container neighbour, circular buffer, and network buffer [27]. This paper used density measure with the “circular buffer” method based on Geographic Information System (GIS). We measured density of different food outlets (the number of target food outlet within buffer around the respondent’s home), based on Euclidean distance (straight line distance). A buffer distance of 1000 m was used.

Data on food outlets was collected by Food and Environmental Hygiene Department (FEHD), Hong Kong [28], which provided information on the addresses and categories for the food restaurants and food stores. The FEHD classified food outlets into 12 categories with three categories of food restaurants (including general restaurant, light refreshment restaurant, marine restaurant) and nine categories of food stores (bakery, cold stores, factory canteen, food factory, fresh provision shop, frozen confection factory, milk factory, siu mei and lo mei shop, and composite food shop) (refer to Table 1 for description of each category) [29]. In this paper, we re-categorized the food outlets (*n* = 15,171) into grocery stores (*n* = 2579), convenience stores (*n* = 9752) and fast-food restaurants (*n* = 2840) based on the classification of FEHD so that it could be compared with findings in North America [17,18,19]. Table 1 summarizes the reclassification. Original categories of general restaurant, marine restaurants, cold stores, and milk factory were excluded in our new classification because general restaurant and marine restaurants could be seen as full service restaurants that sale both health and unhealthy foods; cold stores and milk factory are not direct to its surrounding consumers which distribute goods through logistics. In the new classification, grocery stores were defined as fresh provision shops which involve the sale of fresh, chilled or frozen beef, mutton, pork, reptiles (including live reptiles), fish (including live fish) or poultry (including live poultry), including supermarkets, street market, and other fresh stores. Convenience stores were defined as bakery, factory canteen, food factory, frozen confection factory, siu mei and lo mei shop (siu mei refers to meats roasted on spits over an open fire or a large wood burning rotisserie oven; lo mei refers to pot-stewed food), and composite food shop, which provide limited and convenient goods. Fast-food restaurants were defined as light refreshment restaurants, which sell limited food items.

The address of food outlet was geocoded to obtain latitude and longitude using Google Map API. Density of target food outlet was calculated in ArcGIS 10.4.1 (Esri, Redlands, CA, USA) by the Buffer and the Count Point in Polygon analysis tools. A Retail Food Environment Index (RFEI) [18] was calculated for each respondent based on the following formula: RFEI = (F + C)/G where F represents density of fast-food restaurants; C represents density of convenience stores; and G represents density of grocery stores (including supermarkets, street market and other fresh provision shops). RFEI is a ratio of describing the relative density of unhealthy food outlets to healthy food outlets. A higher RFEI indicates an unhealthier food environment. Thus, totally four food environment measures (density of grocery stores, convenience stores, fast-food restaurants, and RFEI) were used in this paper. All food environment measures were stratified into quartiles for analysis.

### 2.4. Statistical Analysis

Logistic regression model was performed on the association between FV consumption and each food environment measures (quartiles). The dependent variables were infrequent fruit consumption (<7 days/week) and infrequent vegetable consumption (<7 days/week). The independent variables were each food environment measure (density of grocery stores, convenience stores, fast-food restaurants and RFEI) and covariates. Two models were computed for each food environment measure. Model 1 included only food environment measure and no other covariates. Model 2 adjusted for significant covariates by entering all socio-demographic variables and significant covariates were selected by “Backward Elimination (Conditional)” method in SPSS. The significant covariates for association with infrequent fruit consumption included age, gender, marital status, house type, and self-reported poverty level while the significant covariates for association with infrequent vegetable consumption included age, gender, marital status, house type, self-reported poverty level, birth place and whether having under school age children. All statistical analyses were conducted using IBM SPSS 20.0 (SPSS Inc., Chicago, IL, USA). *p*-value < 0.05 were considered significant. Quartile 1 for each food environment measure was used as reference category in the models.

### 2.5. Sensitivity Analysis

We were concerned that different buffer distances and stratified food environment measures might affect the associations between local retail food environment and FV consumptions. Therefore, sensitivity analyses with these two dimensions were performed for infrequent fruit consumption and infrequent vegetable consumption. Buffer sizes included 500 m, 1000 m, 1500 m, 2000 m, and food environment measures were stratified by not only quartiles, but also tertiles and quintiles.

## 3. Results

Among 1977 adults, 7 (0.3%) adults had no grocery stores within 1000 m buffer around their homes. The RFEI cannot be calculated for these individuals since their RFEI was infinite. To include all the participants in the analysis, we replaced 0.1 with 0 for the grocery store variable. These seven adults had Q4 RFEI. Descriptive statistics, overall and stratified by quartiles of RFEI are shown in Table 2. The RFEI were divided into quartiles (Q): Q1 < 2.25, Q2 2.25–3.40, Q3 3.40–5.76, Q4 > 5.76. All respondents (*n* = 1977) in this study are adults aged over 18, with 58.9% female, 57.1% living in public rent housing, 26.7% with low income, 24.1% in self-reported poverty, 85.4% with education attainment under college, 38.2% in single marital status, 48.4% born in Hong Kong and 8.3% having under school-age children. Respondents with highest RFEI (Q4, >5.76) tended to be with low income and older. For example, 30% of respondents with highest RFEI was low income while 23.1% of respondents with lowest RFEI (Q1, <2.25) was low income. 39.3% of respondents with highest RFEI was over 60 years old, while 29.6% of respondents with lowest RFEI was over 60 years old. Neighbourhoods with highest RFEI consisted of lower density of grocery stores and higher density of convenience stores and fast-food restaurants. For instance, neighbourhoods with highest RFEI consisted with a mean (SD) of 40.99 (23.00) grocery stores, 268.23 (135.36) convenience stores and 60.28 (44.20) fast-food restaurants while neighbourhoods with lowest RFEI consisted with a mean (SD) of 41.04 (18.88) grocery stores, 59.85 (29.37) convenience stores, and 15.15 (9.36) fast-food restaurants. The mean (SD) density of grocery stores, convenience stores and fast-food restaurants within 1000 m buffer around respondents’ homes was 43.27 (26.34), 147.05 (123.51), and 40.76 (43.90) separately. Overall, 694 (35.1%) respondents consumed fruit infrequently (<7 days/week) and 187 (9.5%) respondents consumed fruit infrequently (<7 days/week), and this lack of consumption differed by RFEI. Respondents with highest RFEI were more likely to consume fruit (38%) and vegetable (12%) infrequently, relative to those with lowest RFEI (fruit 33%; vegetable 8%).

Table 3 shows associations of four food environment measures with infrequent fruit consumption. The RFEI was positively associated with infrequent fruit consumption. Specifically, the odds of a respondent consuming fruit infrequently were significantly greater (OR = 1.36, 95% CI 1.04–1.78) if they lived in an area with the highest RFEI in comparison to the lowest RFEI after significant covariates adjusted including age, gender, marital status, house type, and self-reported poverty level. The density of fast-food restaurants was also positively associated with infrequent fruit consumption. In the unadjusted model 1, respondents with highest density of fast-food restaurants (Q4, >53) had 1.34 (95% CI 1.04–1.73) times the odds of infrequent fruit consumption compared to those with lowest density of fast-food restaurants (Q1, <13). Adjusting for significant covariates (model 2) attenuated this association (Q4 vs. Q1, OR = 1.29, 95% CI 0.99–1.68).

Table 4 shows associations of four food environment measures with infrequent vegetable consumption. Similar to associations with infrequent fruit consumption, the RFEI was also positively associated with infrequent vegetable consumption. Respondents with the highest RFEI had 1.72 (95% CI 1.11–2.68) times the odds of infrequent fruit consumption compared to those with lowest RFEI after adjusting for significant covariates (including age, gender, marital status, house type, self-reported poverty level, birth place, and whether having under school-age children) in model 2. Overall, higher density of grocery stores was associated with smaller odds of infrequent FV consumption and higher density of convenience stores or fast-food restaurants was associated with greater odds of infrequent FV consumption, however, the association of absolute density of each type of food outlet was not significant, except for the density of fast-food restaurants with fruit consumption.

Table S1 (see Supplementary Materials) shows the sensitivity analyses’ results on odds of infrequent fruit consumption based on four buffer sizes (500 m, 1000 m, 1500 m, 2000 m) and three stratified methods of food environment measures (quartiles, tertiles, and quintiles). Table S2 (see Supplementary Materials) shows the sensitivity analyses’ results on odds of infrequent vegetable consumption. The choice of use of quartiles only affect the impact size (OR value) since OR value is related to the unit of variables. No matter the use of quartiles, tertiles, or quintiles, the significance and direction of associations are very similar. However, the choice of buffer size did affect the significance of associations. The associations between RFEI (quartiles, tertiles, and quintiles) and odds of infrequent fruit consumption were significant with buffer sizes of 1000 m, 1500 m, and 2000 m. However, the associations between RFEI (quartiles, tertiles, and quintiles) and the odds of infrequent vegetable consumption were only significant with the 1000 m buffer.

## 4. Discussion

In this study, we explored whether the density of different types of retail food outlets (grocery stores, convenience stores and fast food restaurants) and the relative density of fast-food restaurants and convenience stores to grocery stores’ RFEI around people’s home was associated with infrequent consumption of fruit or vegetable among adults in Hong Kong. We found significant associations between RFEI and FV consumption after covariates adjusted. The higher the ratio of fast-food restaurants and convenience stores to grocery stores, the greater the odds of infrequent FV consumption. A higher density of fast food restaurants was also significantly associated with greater odds of infrequent fruit consumption in unadjusted model. Although generally higher density of grocery stores was associated with smaller odds of infrequent FV consumption and higher density of convenience stores was associated with greater odds of infrequent FV consumption, the association was not significant regardless of the covariates included in the model. Thus, despite our findings supported mainstream assumptions in western countries that grocery stores were healthy food outlets and fast-food restaurants or convenience stores were unhealthy food outlets, the measure of density of single types of food outlets is not suitable for local food environment assessment in Hong Kong. Instead, the relative density of unhealthy food outlets (i.e., fast-food restaurants and convenience stores) to healthy food outlets (i.e., grocery stores) is an effective indicator for characterizing local retail food environment to predict risk of unhealthy diet in Hong Kong. Such findings may also help explain prevalence of obesity and other food-related chronic diseases including cardiovascular diseases, cancer, and diabetes in Hong Kong [30].

To our knowledge, this study is the first to measure the local retail food environment and examine its association with fruit and vegetable consumption in adult population within a Hong Kong’s context. In our study, the mean (SD) and median of RFEI were 4.61 (4.08) and 3.40 after excluding seven (0.3%) adults who had no grocery stores within a 1000 m buffer around their homes, i.e., the resident in Hong Kong was exposed to approximately 4.61 times more fast-food restaurants and convenience stores within 1000 m of their home than grocery stores which was similar to studies about RFEI in California (the United States) with an average RFEI of 4.5 within a buffer of 800 m for urban residents, 1000 m for smaller cities and suburban areas and 5000 m for rural areas [17] and Edmonton (Canada) with an average RFEI of 5.09 within an 800 m buffer around the residence [18]. These two studies about RFEI assumed that local food environments influence individual eating choice and consequently influence their health. Although they revealed the relation between RFEI and diet-related health outcomes and found that higher RFEI was associated with higher prevalence of obesity and diabetes, both of them did not investigate the association between the local food environment and food consumption pattern. Our findings that RFEI was associated with FV consumption supports these two studies. Specifically, we found that people lived in an area with the highest RFEI (Q4, >5.76) had 1.36 (95% CI 1.04–1.78) times the odds of not eating fruit every day after covariates were adjusted, 1.52 (95% CI 0.99–2.33) times the odds of not eating vegetable every day, and 1.72 (95% CI 1.11–2.68) times after covariates were adjusted in comparison to the lowest RFEI (Q1, <2.25).

From these results, we also can notice the reinforcement of the associations between the relative food retailer index and FV consumption when significant covariates were entered in our analysis. For example, the odds of infrequent vegetable consumption for the highest RFEI was increased 13% with the inclusion of age, gender, marital status, house type, self-reported poverty level, birth place, and whether having under school age children in the second model. Besides, the associations between RFEI groups and infrequent FV consumption (<7 days/week) was not significant before adjusting for age, gender, marital status, house type, and self-reported poverty level. Thus, the real obesogenic effect of local unhealthy retail food environment was easily to be neglected if we do not put it into an ecological model [31] which provides a useful framework for a better understanding of the multiple factors of health behaviour. Dietary behaviour, including FV consumption, is influenced by many factors, such as income, food prices (which affect the availability and affordability of healthy foods), individual preferences and beliefs, culture traditions, as well as geographical, environmental, social, and economic factors [1]. The significant determinants of FV consumption included in our study were similar to results of a review by Kamphuis et al. [32] that single people compared with married, people with lower income, who not having children and people surrounding by low food accessibility, had lower FV intake.

Our findings also suggest “food swamps” (areas with an abundance of unhealthy foods) rather than “food deserts” (areas where there is limited access to healthy foods) seem to be more problematic in Hong Kong’s urban areas. Compared to those reported in the U.S., U.K., and Canada studies [33,34,35], we observed Hong Kong had a much higher density of healthy food outlets, but also a higher density of unhealthy food outlets. For instance, the resident in Hong Kong is exposed to an average of 43.27 healthy food outlets (i.e., grocery stores including supermarkets, street markets, and other fresh provision shops) within a 1000 m of their home, and the average density of unhealthy outlets is 187.81, with 147.05 of convenience stores and 40.75 of fast-food restaurants. In New York City, the mean density per square kilometre of healthy food outlets (including supermarket, fruit and vegetable stores, natural/health food stores) was 4.27, and the mean density of unhealthy food outlets (including fast-food restaurants, pizza restaurants, convenience stores, bodegas, bakeries, candy and nut stores, meat markets) was 30.81 [36]. Additionally, in our study, 0.3% of Hong Kong’s adults had no grocery stores within a 1000 m buffer around their homes while, in California, 28% of adults have no grocery stores or produce vendors within the buffer around their homes [17]. The high density of food retailers in Hong Kong could be due to its high population density and compact land use. In Hong Kong, the average population density is about 6800 persons/km^2^ at end-2015 and is about 27,330 persons/km^2^ if only the built-up area is accounted. Thus, controlling neighbourhood unhealthy food retailers could be the primary task for promoting a healthy food environment in the high-density context of Hong Kong.

Strengths of the study include the large representative sample with detailed individual information in Hong Kong and the use of objective GIS-based measures for characterizing local retail food environment with different types of food outlets. Our work attempts to classify all opportunities to obtain food (including food stores and restaurants) and provide a tool that can capture the entire food environment rather than focus on specific types of outlet. Additionally, the FEHD food classification system in Hong Kong is different from the North American Industry Classification System used in the United States, Canada, and Mexico [37], and other classification systems [38]. To make it comparable with findings in North America, we re-classified the food outlets into three types: grocery stores, convenience stores, and fast-food restaurants based on the categories of FEHD. In the new classification, grocery stores mainly provide fresh food, e.g., fresh, chilled or frozen beef, mutton, pork, reptiles (including live reptiles), fish (including live fish), or poultry (including live poultry). Convenience stores and fast-food restaurants provide limited and convenient foods, e.g., frozen foods, siu mei, lo mei, hot dogs, hamburgers, instant noodles, toast, and so on, which are high-sugar or high-fat foods. The results in this study suggest our new classification about food outlets in Hong Kong is feasible. The new food classification we developed could be used by other researchers in Hong Kong to build a picture of the food environment. Furthermore, the sensitivity analysis based on different buffer distances (500 m, 1000 m, 1500 m, 2000 m) is another strength. The buffer distance, often selected on the basis of estimations of neighbourhood walkability or distance that individuals might be ready to cover to reach food outlets, the values used in previous studies varied from 100 to 2000 m [8,12,39]. In our study, among these four buffer distances, only with the 1000 m buffer were the associations between RFEI and FV consumptions significant. This finding could be explained by 1000 m being near the average walking distance for shopping and eating in Hong Kong. The 2011 Travel Characteristics Survey in Hong Kong [40] showed that the mean waking trip time for Home-Based Others (from home to places that are not usual work place or schools, e.g., shopping places, food premises, entertainment places, visiting friends, etc.) in the Hong Kong population was nine minutes (around 800–1000 m). Therefore, a 1000 m buffer could be a reasonable distance in Hong Kong’s context.

Several limitations of the study should be considered. First, the present data is based on a cross-sectional design from which it is not possible to determine the causal relation between food environment and dietary behaviour outcomes. Second, sensitivity analyses for other set parameters for GIS-based measures (e.g., access approach, distance type, and buffer type) should be further studied since these might result in mixed findings [10]. Thirdly, Glanz et al. [6] identified two aspects of the food environment, one is the community nutritional environment defined by proxy measures, such as the number, type, location, and accessibility of food outlets, and another is the consumer nutritional environment, defined by directly-measured food availability in and around food outlets (e.g., prices, promotions, and nutritional quality). In the present study, we have considered the community nutritional environment. More comprehensive and multi-source data about food outlets are needed for a better theoretical understanding of individual eating choices. Finally, although our study suggests that respondents with highest RFEI (Q4, >5.76) tended to be with low income and older populations, we did not explore the mediating and moderating pathways among individual characteristics and the local food environment. Additionally, the distribution of the local retail food environment could be associated with neighbourhood characteristics, especially socioeconomic status, which contribute to the disparities in food access [33]. Moreover, the associations of the built environment, including the food environment and physical activity environment, with diet-related health outcomes such as obesity or other chronic diseases were not explored in Hong Kong. Therefore, a series of research about food environment in Hong Kong are needed to help a better understanding of the complex effect of environment on health and to provide an evidence base for promoting a healthy food environment.

## 5. Conclusions

In summary, the current study was the first to provide evidence for the influence of the local retail food environment on the dietary behaviour, i.e., the consumption of fruit and vegetable in Hong Kong’s setting using GIS-based methods. Additionally, it was among the few to document such associations of density of different types of retail food outlets with fruit and vegetable consumption outside of the western societies. Our results suggest that “food swamps” (areas with an abundance of unhealthy foods) rather than “food deserts” (areas where there is limited access to healthy foods) seems to be more of a problem in Hong Kong’s urban areas. Importantly, our results suggest that the ratio of fast-food restaurants and convenience stores to grocery stores near people’s home is critical for meeting fruit and vegetable consumption guidelines and is an effective, objective indicator for characterizing the local retail food environment in Hong Kong. Therefore, improving the local retail food environment by zoning regulation which limits the density of fast-food restaurants or convenience stores and increases the density of fresh food stores seems to be a feasible policy option for promoting a healthy diet among adults. Research on the practical effectiveness of such interventions is needed.

## Figures and Tables

**Table 1:** Reclassification of retail food outlets into three types derived from FEHD.

Reclassification of Retail Food Outlets into Three Types	Original Classification of Retail Food Outlets by FEHD *	Description
Grocery store	Fresh provision shop	Involves the sale of fresh, chilled or frozen beef, mutton, pork, reptiles (including live reptiles), fish (including live fish) or poultry (including live poultry), but does not include a restaurant, factory canteen, market stall, or any business carried on by a hawker who is the holder of a licence under the Hawker Regulation
Convenience store	Bakery	Involves the baking of bread and other bakery products for sale
	Cold stores (excluded)	Involves the storage of articles of food under refrigeration in any warehouse in Hong Kong
	Factory canteen	Any food business in a factory building which involves the sale or supply of meals or unbottled non-alcoholic drinks other than Chinese herb tea for consumption on the premises by persons employed in any factory in that factory building
	Food factory	Involves the preparation of food for sale for human consumption off the premises, but does not include a frozen confection factory, a milk factory or any business carried on by a hawker who is the holder of a licence under the Hawker Regulation
	Frozen confection factory	Involves, within the meaning of the Frozen Confections Regulation, the manufacture of any frozen confection in the territory
	Milk factory (excluded)	Involves, within the meaning of the Milk Regulation, the processing or reconstitution of milk or any milk beverage in the territory
	Siu mei and Lo mei shop	Involves the sale by retail of siu mei or lo mei, but does not include a restaurant, factory canteen, or any business carried on by a hawker who is the holder of a licence under the Hawker Regulation
	Composite food shop	Covers the sale and preparation for sale of various specified types of simple or ready-to-eat foods that do not involve complicated preparation
Fast-food restaurant	Light refreshment restaurant	This licence restricts the licensee to prepare and sell for consumption on the premises any one group of the food items listed in Appendix B * of A Guide to Application for Restaurant Licence.
	Marine restaurant (Excluded)	Operation of restaurant business on board a vessel
	General restaurant (Excluded)	Sell any kind of food for consumption

FEHD: Food and Environmental Hygiene Department; The FEHD classified food outlets into 12 categories with 3 categories of food restaurants (including general restaurant, light refreshment restaurant, marine restaurant) and 9 categories of food stores (bakery, cold stores, factory canteen, food factory, fresh provision shop, frozen confection factory, milk factory, siu mei and lo mei shop and composite food shop). We excluded the general restaurant, marine restaurants, cold stores and milk factory when reclassified retail food outlets. Details about description of FEHD’s category could be seen in https://www.fehd.gov.hk/english/licensing/Guide_on_Types_of_Licences_Required.html. * Appendix B: www.fehd.gov.hk/english/howtoseries/forms/new/A_Guide_to_Restaurant.PDF.

**Table 2:** Characteristics of the study sample (*n* = 1977) by Retail Food Environment Index (RFEI) Quartiles.

	Retail Food Environment Index (RFEI) Quartile (Q) ^a^				All, *n* = 1977
	Q1, *n* = 494 (<2.25)	Q2, *n* = 496 (2.25–3.40)	Q3, *n* = 483 (3.40–5.76)	Q4, *n* = 504 (>5.76)	
Gender (*n* (%))					
Female	284 (57.5)	295 (59.5)	289 (59.8)	296 (58.7)	1164 (58.9)
Male	210 (42.5)	201 (40.5)	194 (40.2)	208 (41.3)	813 (41.1)
Age (*n* (%))					
18–39	119 (24.1)	131 (26.4)	115 (23.8)	112 (22.2)	477 (24.1)
40–59	229 (46.4)	191 (38.5)	190 (39.3)	194 (38.5)	804 (40.7)
60+	146 (29.6)	174 (35.1)	178 (36.9)	198 (39.3)	696 (35.2)
House type (Public Rent Housing) (*n* (%))					
Non-public rent housing	136 (27.5)	266 (53.6)	230 (47.6)	216 (42.9)	848 (42.9)
Public rent housing	358 (72.5)	230 (46.4)	253 (52.4)	288 (57.1)	1129 (57.1)
Monthly income (*n* (%))					
Not low income	380 (76.9)	372 (75)	344 (71.2)	353 (70)	1449 (73.3)
Low income (<HK$ 3500)	114 (23.1)	124 (25)	139 (28.8)	151 (30)	528 (26.7)
Self-reported poverty b (*n* (%))					
No	356 (72.1)	389 (78.4)	373 (77.2)	382 (75.8)	1500 (75.9)
Yes	138 (27.9)	107 (21.6)	110 (22.8)	122 (24.2)	477 (24.1)
Education attainment (*n* (%))					
Higher education	58 (11.7)	74 (14.9)	81 (16.8)	76 (15.1)	289 (14.6)
Education attainment under college	436 (88.3)	422 (85.1)	402 (83.2)	428 (84.9)	1688 (85.4)
Marital status (*n* (%))					
Non-single	321 (65)	308 (62.1)	280 (58)	312 (61.9)	1221 (61.8)
Single	173 (35)	188 (37.9)	203 (42)	192 (38.1)	756 (38.2)
Birth place (*n* (%))					
Non-Hong Kong	262 (53)	249 (50.2)	239 (49.5)	270 (53.6)	1020 (51.6)
Hong Kong	232 (47)	247 (49.8)	244 (50.5)	234 (46.4)	957 (48.4)
Having under school age children (*n* (%))					
Yes	41 (8.3)	45 (9.1)	36 (7.5)	43 (8.5)	165 (8.3)
No	453 (91.7)	451 (90.9)	447 (92.5)	461 (91.5)	1812 (91.7)
Density of food outlets (mean (SD))					
Grocery stores	41.04 (18.88)	37.96 (24.51)	53.37 (34.19)	40.99 (23.00)	43.27 (26.34)
Convenience stores	59.85 (29.37)	85.26 (56.82)	173.24 (107.03)	268.23 (135.36)	147.05 (123.51)
Fast-food restaurants	15.15 (9.36)	23.38 (20.41)	64.43 (58.32)	60.28 (44.20)	40.76 (43.90)
Fruit consumption (*n* (%))					
Infrequent fruit consumption (<7 days/week)	165 (33)	148 (30)	188 (39)	193 (38)	694 (35.1)
Frequent fruit consumption (7 days/week)	329 (67)	348 (70)	295 (61)	311 (62)	1283 (64.9)
Vegetable consumption (*n* (%))					
Infrequent vegetable consumption (<7 days/week)	39 (8)	43 (9)	47 (10)	58 (12)	187 (9.5)
Frequent vegetable consumption (7 days/week)	455 (92)	453 (91)	436 (90)	446 (88)	1790 (90.5)

^a^ Retail Food Environment Index (RFEI) is calculated by dividing the total number of fast-food restaurants and convenience stores by the total number of grocery stores (including supermarkets, street market and other fresh provision shops) within 1000 m of the respondent’s home based on Euclidean distance. Quartile (Q): Q1 = quartile with lowest RFEI–Q4 = quartile with highest RFEI. ^b^ Self-reported poverty was surveyed by question, “Do you think you are poor now?”.

**Table 3:** Associations between local food environment and odds of infrequent fruit consumption (<7 days/week) among adults in Hong Kong with binary logistic regression models.

	Quartile (Range)	Model 1 ^a^	Model 2 ^b^
		OR (95% CI)	OR (95% CI)
Retail Food Environment Index (RFEI)	Q1 (<2.25)	Ref	Ref
	Q2 (2.25–3.40)	0.85 (0.65, 1.11)	0.94 (0.71, 1.24)
	Q3 (3.40–5.76)	1.27 (0.98, 1.65)	1.40 (1.07, 1.84) *
	Q4 (>5.76)	1.24 (0.96, 1.60)	1.36 (1.04, 1.78) *
Density of grocery stores	Q1 (<20)	Ref	Ref
	Q2 (20–44)	1.06 (0.82, 1.38)	0.97 (0.74, 1.28)
	Q3 (44–60)	0.93 (0.71, 1.22)	0.81 (0.61, 1.08)
	Q4 (>60)	1.00 (0.77, 1.29)	0.87 (0.66, 1.15)
Density of convenient stores	Q1 (<58)	Ref	Ref
	Q2 (58–100)	0.89 (0.68, 1.15)	0.80 (0.60, 1.05)
	Q3 (100–222)	0.92 (0.71, 1.20)	0.87 (0.67, 1.14)
	Q4 (>222)	1.19 (0.92, 1.54)	1.11 (0.85, 1.45)
Density of fast-food restaurants	Q1 (<13)	Ref	Ref
	Q2 (13–25)	1.07 (0.83, 1.39)	0.92 (0.70, 1.21)
	Q3 (25–53)	1.14 (0.88, 1.48)	1.06 (0.81, 1.39)
	Q4 (>53)	1.34 (1.04, 1.73) *	1.29 (0.99, 1.68)

Density of food outlets was calculated by the number of target food outlet within 1000 m Euclidean buffer of the respondent’s home. All food environment measures were divided into quartiles; * *p* < 0.05; ^a^ Model 1 is an unadjusted model; ^b^ Model 2 adjusts for significant covariates: age, gender, marital status, house type and self-reported poverty level. Ref: reference category.

**Table 4:** Associations between local food environment and odds of infrequent vegetable consumption (<7 days/week) among adults in Hong Kong with binary logistic regression models.

	Quartile (Min–Max)	Model 1 ^a^	Model 2 ^b^
		OR (95% CI)	OR (95% CI)
Retail Food Environment Index (RFEI)	Q1 (<2.25)	Ref	Ref
	Q2 (2.25–3.40)	1.11 (0.70, 1.74)	1.26 (0.79, 2.02)
	Q3 (3.40–5.76)	1.26 (0.81, 1.96)	1.39 (0.88, 2.20)
	Q4 (> 5.76)	1.52 (0.99, 2.33)	1.72 (1.11, 2.68) *
Density of grocery stores	Q1 (<20)	Ref	Ref
	Q2 (20–44)	1.14 (0.75, 1.73)	0.99 (0.64, 1.55)
	Q3 (44–60)	0.89 (0.57, 1.40)	0.77 (0.48, 1.24)
	Q4 (>60)	1.05 (0.69, 1.61)	0.90 (0.58, 1.42)
Density of convenient stores	Q1 (<58)	Ref	Ref
	Q2 (58–100)	1.07 (0.68, 1.68)	0.98 (0.61, 1.57)
	Q3 (100–222)	1.28 (0.83, 1.97)	1.23 (0.79, 1.92)
	Q4 (>222)	1.39 (0.91, 2.12)	1.32 (0.85, 2.06)
Density of Fast-food restaurants	Q1 (<13)	Ref	Ref
	Q2 (13–25)	1.17 (0.76, 1.79)	0.94 (0.60, 1.48)
	Q3 (25–53)	1.19 (0.78, 1.81)	1.11 (0.71, 1.73)
	Q4 (>53)	1.12 (0.73, 1.70)	1.03 (0.66, 1.61)

Density of food outlets was calculated by the number of target food outlet within 1000 m Euclidean buffer of the respondent’s home. All food environment measures were divided into quartiles; * *p* < 0.05; ^a^ Model 1 is an unadjusted model; ^b^ Model 2 adjusts for significant covariates: age, gender, marital status, house type, self-reported poverty level, birth place, and whether having under school age children. Ref: reference category.

## Supplementary Materials

The following are available online at http://www.mdpi.com/1660-4601/15/10/2247/s1; Table S1. Sensitivity analysis on the odds of infrequent fruit consumption based on different buffer sizes and stratified food environment measures; Table S2. Sensitivity analysis on odds of infrequent vegetable consumption based on different buffer sizes and stratified food environment measures.
